# Experiences of a commercial weight-loss programme after primary care referral: a qualitative study

**DOI:** 10.3399/bjgp15X684409

**Published:** 2015-03-30

**Authors:** Jodie T Allen, Simon R Cohn, Amy L Ahern

**Affiliations:** The Primary Care Unit, Institute of Public Health, University of Cambridge, Cambridge.; Department of Health Services Research and Policy, London School of Hygiene and Tropical Medicine, London, and senior lecturer, The Primary Care Unit, Institute of Public Health, University of Cambridge, Cambridge.; MRC Human Nutrition Research, Elsie Widdowson Laboratory, Cambridge, Cambridge.

**Keywords:** general practice, obesity, primary health care, qualitative research, referral and consultation, weight loss

## Abstract

**Background:**

Referral to a commercial weight-loss programme is a cost-effective intervention that is already used within the NHS. Qualitative research suggests this community-based, non-medical intervention accords with participants’ view of weight management as a lifestyle issue.

**Aim:**

To examine the ways in which participants’ attitudes and beliefs about accessing a commercial weight management programme via their doctor relate to their weight-loss experience, and to understand how these contextual factors influence motivation and adherence to the intervention.

**Design and setting:**

A qualitative study embedded in a randomised controlled trial evaluating primary care referral to a commercial weight-loss programme in adults who are overweight or obese in England. The study took place from June–September 2013.

**Method:**

Twenty-nine participants (body mass index [BMI] ≥28 kg/m^2^; age ≥18 years), who took part in the WRAP (Weight Loss Referrals for Adults in Primary Care) trial, were recruited at their 3-month assessment appointment to participate in a semi-structured interview about their experience of the intervention and weight management more generally. Interviews were audiorecorded, transcribed verbatim, and analysed inductively using a narrative approach.

**Results:**

Although participants view the lifestyle-based, non-medical commercial programme as an appropriate intervention for weight management, the referral from the GP and subsequent clinical assessments frame their experience of the intervention as medically pertinent with clear health benefits.

**Conclusion:**

Referral by the GP and follow-up assessment appointments were integral to participant experiences of the intervention, and could be adapted for use in general practice potentially to augment treatment effects.

## INTRODUCTION

Health risks associated with obesity are well established; the 2007 Foresight report estimates that, by 2050, obesity-related illness could cost the UK NHS over £9 billion per year.^1^ In May 2014, the National Institute for Health and Care Excellence (NICE) published guidelines recommending that primary care practitioners use behavioural weight management programmes as a first-line intervention for adults who are overweight or obese.^2^

Although many patients who are overweight would welcome more assistance with weight management from their GP, by positioning causality and treatment of obesity as an issue of lifestyle choices and individual responsibility, both health professionals and patients generally perceive weight management as falling outside the GP’s remit.^3^–^6^ Qualitative research also suggests that, by delivering the interventions in the community, commercial weight management programmes align better with participants’ explanatory model of obesity and expectations regarding appropriate treatment.^4^ This is supported by evidence suggesting GP-led interventions have limited effectiveness in helping people lose weight, and that commercial programmes are more clinically and cost effective.^7^–^10^

In the UK, Public Health and NHS commissioners are currently able to purchase 12-week referral packages from a number of commercial weight-loss providers at a reduced cost. GPs and other health professionals are thereby able to provide their patients with vouchers to attend meetings and use internet-based resources. However, there is some scepticism among patients about the profit-making interests of commercial companies and concern over the public funding of weight-loss services.^5^

This study draws on interviews with participants from a randomised controlled trial (RCT) of primary care referral to a commercial weight-loss programme. It examines their attitudes and beliefs about accessing a commercial weight-loss programme via their doctor, and the ways in which these relate to their weight-loss experience. This study explores their views about an NHS commercial partnership and the provision of ‘free’ weight management, paid for by the NHS, in order to understand the contextual factors that may facilitate participation in the programme.

## METHOD

### Design and setting

This qualitative study was embedded within the WRAP Weight Loss Referrals for Adults in Primary Care) (ISRCTN82857232) randomised controlled trial, the full protocol for which is published elsewhere.^11^ The trial recruited adults who were overweight or obese and randomised them to either: referral to a commercial programme (Weight Watchers^®^) for 12 weeks; referral to the same programme for 52 weeks; or a brief self-help intervention. Clinical measures (weight, waist circumference, body composition, and blood pressure) and self-report questionnaires were completed at baseline, 3, 12, and 24 months.

**How this fits in**

Randomised controlled trials have shown that referral to a commercial weight-loss programme is a cost-effective intervention for people who are overweight and obese, and qualitative research has shown that it accords with patients’ views of weight management as a non-medical lifestyle issue. This study suggests that patients do not see a clear demarcation between the primary care and commercial sectors, and that the referral from the GP and subsequent clinical assessments (undertaken as part of a randomised controlled trial) frame patient experience of this community-based intervention to strengthen the focus on health benefits. The perceived investment and medical observation from the GP may increase motivation of patients to attend the commercial programme and engage in weight management. Short follow-up appointments, with clinical measurements fed back to the GP, could be incorporated into clinical practice to potentially enhance attendance, adherence, and sustain intervention effects for patients referred to commercial programmes.

### Recruitment and sampling

For the main trial, participating GP practices used their lists to identify all eligible males and females, (aged ≥18 years with a body mass index [BMI] >28 kg/m^2^) and sent them invitations to participate. In total, 1269 participants were recruited by three research centres (MRC Human Nutrition Research, Cambridge; the University of Oxford; and the University of Liverpool) from 23 GP practices across the UK.

A purposeful sample of 29 participants from the Cambridge centre participated in this qualitative study. A maximum-variation (heterogeneity) sampling technique was used to select potential interviewees based on BMI, intervention arm, and demographic information (age, sex, ethnicity, and education) obtained during the telephone screening questionnaire and through a questionnaire at the baseline visit. At the 3-month visit, one author met with selected participants (including some who had stopped attending meetings but not withdrawn from follow-up) and invited them to participate in the qualitative study. Interested participants were given a participant information sheet to take home with them and were asked to contact the author if they were happy to take part in an interview.

Participants could choose whether to be interviewed in their home or in a private office at the University of Cambridge. Interviews were not held where study procedures were conducted. This was to reduce associations between the interview and the measurement visits of the trial to encourage participants to speak openly about their experiences of the intervention to which they had been assigned. The interviewer told participants that she worked alongside the trial but was not part of the core trial team.

### Data collection and analysis

Face-to-face semi-structured home interviews with participants were conducted by one author between June and September 2013. The interview focused on patients’ experiences, attitudes, and beliefs concerning:

- the GP referral process to the commercial programme;
- their participation in the commercial programme; and
- the idea of being overweight as a ‘medical’ issue.

Interviews were audiorecorded, transcribed verbatim, and analysed inductively using a narrative approach.^12^ In this way, although the interviews followed a common topic guide, the structure generated first-order descriptive codes that were augmented through a process of inductive analytic coding to identify the major underlying themes.^13^

Data were coded by one author using NVivo (version 10); the first five interviews were coded blind by all authors to ensure consistency and appropriateness of categories. All authors are experienced qualitative researchers, but from different disciplinary backgrounds. Regular discussion and reflection thereby ensured that codes and coding were achieved through consensus.

## RESULTS

Participant characteristics are described in Table 1. Five main topics emerged from analysis of the data that address the core research question.

**Table 1.** Participant characteristics recorded at the baseline appointment. BMI was measured by research staff and all other characteristics are self-reported

	**CP12**	**CP52**	**Brief intervention**	**Total**
**Sex**				
Male	5	4	0	9
Female	7	8	5	20

**Age, years**				
<40	4	5	0	9
40–49	2	3	2	7
50–59	3	1	1	5
≥60	3	3	2	8

**BMI, kg/m^2^**				
< 30	2	1	0	3
30–34.9	6	6	2	14
35–39.9	3	2	2	7
≥40	1	3	1	5

**Ethnicity**				
Asian/Asian British	1	1	0	2
Black/black British	0	0	0	0
Mixed/multiple ethnic groups	1	2	0	3
White British	9	7	5	20
White other	1	2	0	3

**Education**				
Higher degree	3	4	0	7
University degree	5	0	2	7
A Level or equivalent	2	3	1	6
GCSE or equivalent	2	1	0	3
Other (vocational)	0	2	2	4
Not stated	0	2	0	2

BMI = body mass index. CP12 = 12-week commercial programme. CP52 = 52-week commercial programme.

### Accounts of usual primary care

Patients for whom weight loss had been raised as a topic during a GP consultation reported that they received little to no practical support, for example stating:

‘*You’re kind of left to your own devices to deal with it.*’

(Female, 61)

‘*I had a problem with my back and the most useful thing* [the doctor]*, could come up with was some pain medicine and say “You’re too big, think about losing some weight”, which is fair as a comment but then nothing came to say, you know, “This is who you could talk to to get some help and some information” … anything practical was lacking … If I go to the doctor because I’ve broke my arm they’re not going to tell me “You broke your arm, go get it bandaged up”, they’re going to do something about it or at least tell me what to do about it … I found that a bit annoying at the time.*’

(Female, 32)

Many said that although they had been given leaflets these were inadequate and contained outdated or incorrect information, further cementing their perception that the GP was not invested in providing weight-loss support:

‘*I think first of all she gave me a leaflet which I took home and I showed it to my son and my son said “What the hell? Ah!” And he pointed out all the wrong things, like it said you could eat dried fruit and he said “Dried fruit is full of sugar!”* [patient has type 2 diabetes]. *He was pointing out all the discrepancies and I pointed it out to* [the doctor] *and she said “Hmm, the leaflets are a bit old”* [laughs].’

(Female, 53)

Despite these general feelings, patients did not regard the GP as the appropriate person to help them. Although patients expressed some concern about the health consequences of excess weight, they did not view being overweight or obese as primarily medical issues, and therefore felt this should not become a burden on their GP or the NHS. They consequently welcomed what they saw as referral to more appropriate support beyond the health service.

### The GP letter

Patients were recruited to the trial by their GP, who sent a signed letter to eligible patients in the practice. In this letter, patients were asked to contact the research team if they wished to participate. All subsequent contact regarding the trial, including all measurement visits, was made by the research team. Nevertheless, patients welcomed what they interpreted to be a personal invitation from their doctor, and they frequently mentioned this letter rather than any of the following communications. Although the support was overtly through an existing commercial programme, the sense of personalised support from the GP was enduring. Patients viewed the letter as evidence that they had been specially ‘*nominated*’ (female, 61) or ‘*selected*’ (female, 48) by their GP, who ‘doesn’t offer it [the programme] *to everyone*’ (female, 36). This motivated patients not only to sign up to the programme but also to attend meetings:

*‘I thought my doctor’s helping me, she’s not just sitting back and going “Just get on with it”, you know, … if you just done it on your own you’ve got no support or anything really, you’re just plodding on every week … I’ve got nobody to say that to. But this is why it’s really pushing me to do this and I can go into my doctor’s office and she’ll go “God haven’t you lost weight* [name of participant]*”. You know, because I’m really friendly with my doctor, obviously because I go there every blinking month. So I want to do it as well so she can say to me, “God you are really getting your act together now”, you know, I’m getting rid of this weight.*’

(Female, 70)

In addition, receiving such communication from their doctor framed their weight as a serious issue:

*‘It’s not like I didn’t know or don’t know that I’m much too big but it’s not something that people normally bring up. It just seemed like well if my doctor starts sending me letters … now might be a good time.*’

(Female, 32)

Overall, patients equated the letter from their GP with a more typical NHS referral letter from primary to secondary care, and recognised it as being the key means to access specialist resources and services. As a result, the letter played an important symbolic role for participants.

### The significance of receiving vouchers

Patients viewed the vouchers, which are used to pay for weekly meetings, as a further symbolic link between the commercial programme, their GP, and the NHS. Although the vouchers were given to patients by the research team, patients interpreted them as originating from what they understood to be their GP’s referral. With this came a sense of moral and financial obligation to the GP to attend the commercial programme meetings:

*‘It would be rather embarrassing to be perfectly honest to go to a weight-loss thing and say “Yeah* [bangs table for emphasis] *thank you for the free Weight Watchers things but I just didn’t bother!” … I don’t want to let the doctors down.*’

(Male, 38)

Because participants felt that their weight was caused by ‘*Self-indulgence*’ (Female, 62), and was ‘*... essentially* [their] *fault.*’ (Female, 34), they frequently expressed guilt about drawing on NHS resources to address their problem:

*‘GPs have got sick people to see rather than people who just eat too much.*’

(Female, 46)

In this context, participants welcomed the use of vouchers to access a commercial programme because they would not be ‘*Wasting* [the GP’s] *time*’ (Female, 53), and because they believed it would cost less than the medical interventions that they associated with their problem (for example, bariatric surgery). Vouchers were therefore seen as a means to ensure funding was not taken from the ‘deserving’ sick, whom were regarded as suffering from ‘real’ diseases:

*‘Because the NHS is strapped for cash, there’s people that have got far more serious issues than the fact that I like eating chocolate and crisps, like you’ve got people dying of cancer that they can’t even save, there isn’t enough money available … I would consider that far more serious, because … a lot of cancers are not preventable … so my weight, my weight gain is preventable if I could sort myself out and do it, so I wouldn’t expect someone else to pay for me to get a grip basically* [laughs].’

(Female, 34)

It is worth noting that although, in many accounts, use of the vouchers enabled participants to talk of attending the weight-loss programme ‘for free’, the majority acknowledged that the service was being paid for, and they therefore felt a sense of debt:

*‘I know I’m going to Weight Watchers each week, and I’m not going to let myself down and I’m not letting everybody else down, you know ‘cos it’s costing money, not for me personally but for everybody else, you might as well save the taxpayer, you know, and it’s as simple as that.*’

(Female, 70)

Feeling obliged to use the vouchers because they were free (to them) was contrasted with the idea that if they had paid for the service themselves this would have given them the right not to attend:

*‘I thought I can’t mess people around, if I had decided to do* [the programme] *myself it was very possible that I would have done exactly what other people did* [when] *joining gyms, which is to pay and not go, and I think well it’s alright …* [laughs] *at least if I pay I don’t have to go ... I would have felt I’d done enough in paying and it would have confirmed all my worst feelings about myself, but because I felt that I would be wasting a valuable resource I’ve felt entirely motivated to do those first visits.*’

(Female, 59)

These findings reveal how the vouchers were perceived to be more than a substitute means for payment, and symbolically functioned to blur the demarcation between the NHS — considered a free health service but with limited resources — and a commercial programme.

### The value of clinical measurements

Although participants attending the commercial programme were weighed by the group leader when they attended meetings, they attributed very different meanings to measurements conducted by the research team at both the baseline and 3-month assessment appointments. These measurements were not only regarded as more accurate, but were invariably contextualised by what the participants felt was a clinical framing.

Although all participants acknowledged that they were overweight, the vast majority with a BMI >30 kg/m^2^ did not recognise themselves as being obese. In contrast to themselves, participants described obese individuals in extreme terms such as ‘*elephantine*’, (Male, 63), ‘*housebound and* [needing] *someone else to wash them*’, (Male, 63), unable ‘*to get out of bed ... or go to the toilet*’, (Female, 34), ‘*like* [you see on] *these American* [television] *programmes’,* (Female, 59).

Unlike the term ‘overweight’, patients associated ‘obesity’ with what they considered were far more serious health consequences. Therefore they expressed surprise when they were told that their weight would classify them as obese. As a result, some reported feeling more motivated to lose weight because their problem was suddenly one associated with ill health:

*‘So when I went to the first meeting of the experiment they showed me the chart and said “You’re here and this is for obese”* [laughs] *… before I thought I would be in the overweight category … I definitely do not want to be in the obese category … I guess you are in an unhealthy place … whereas if I was only in the overweight category … it would lack the fact that I’m — I was surprised … that I’m not somewhere where I want to be, … I’m so out of control that I don’t even know that I’m in a bad place … so that was for me more motivational … because I’m so far on the chart, so far beyond what I thought I was, so not in control.*’

(Male, 28)

Waist circumference, body fat percentage, and in some cases blood pressure and BMI were experienced as relatively novel and additional forms of measurement that provided a further ‘shock’ factor not experienced when simply measuring body weight:

‘ [The weight charts] *are the same that I’ve seen many times over really, there was no surprise to me …* ‘*cos you know I’m a serial weight loser and putter oner* [laughs] *so I’ve seen it all … The only thing I was interested in finding out was the percentage of my body fat because I’ve never had any means of doing that … that was a bit shocking ‘cos I’ve got a lot of body fat.*’

(Female, 34)

Tied to an explanation of reducing one’s risk factors for disease, and extending to other ideas they held about the body and health, these measurements gave meaning to weight loss. Patients reported that their previous attempts to lose weight by attending a commercial weight-loss service had been driven more by appearance, and usually precipitated a special occasion or a holiday, and that they felt more committed to losing weight for measurable health improvements:

*‘When I got to the last few weeks before I knew I was coming back to be weighed, the only thing I really could focus on or think about was that I wanted to know my circumference around my waist …* [the researcher] *said “You’ve lost 15 centimetres which means you’re out of the danger zone, you’ve reduced to negligible your chance of getting diabetes”, that one fact was much more easy for me to digest … and hold on to. I have lost weight and now I can see for myself all of the benefits … So that really helps to sort of tie it down to something that’s very, very tangible.*’

(Female, 48)

The measurement visits conducted by the research team provided participants with a new visual language concerning weight loss. For example, they spoke about wanting to move out of the ‘*danger zone*’ or ‘*dark red part of the graph.*’ (Female, 48).

*‘Putting it on more of a chart, my blood pressure and that, … it’s helped me* [because] *I saw the health benefits of it … it reminds me what I’m doing* [and] *how it’s improved.*’

(Female, 37)

By seeing their position on charts, participants felt that what it means to be ‘healthier’ was made more concrete and they expressed a sense of accomplishment from seeing their position alter.

### Attending the commercial programme as a ‘patient’ and trial participant

Some participants reported that they had not attended a commercial programme before because they thought that the ‘*target audience*’ were people who wished to lose weight to improve their ‘*appearance’.* (Male, 38).

Participants considered usual attendance at the commercial programme as more of a leisure activity, which, in a context of limited financial resources, was often viewed as a luxury rather than a necessity:

*‘I’d always put my family first and think, you know, we need that* [money] *somewhere else … I’d feel guilty that again my family is suffering because of my weight, and then they’d go without again because of my problem.*’

(Female, 37)

Men also communicated gendered perceptions of the commercial programme as a ‘*knitting club*’ or ‘*Women’s Institute type of situation.*’ (Male, 51). However, within the trial, the GP invitation to the commercial programme not only served to redefine weight loss as being a medical goal for those who previously thought their problems were primarily aesthetic, but also endorsed the programme as suitable for those who already wanted to lose weight for health reasons.

Patients were also motivated by the fact that they were attending meetings with people who, unlike them, were paying to attend:

*‘I need to embrace this opportunity, 12 weeks free, because it is expensive to go, you know, £5 a week … my friend joined about a week before I did it.*’

(Female, 37)

*‘I feel a bit guilty to them ‘cos … they’re paying to come here.*’

(Female, 34)

The vouchers, provided in an initial 12-week pack to those randomised to both commercial programme interventions, served to make explicit the time commitment required. This was contrasted with the open nature of commitment for non-referral attendees, who, instead of time, are encouraged to pursue a weight-loss goal.

Although those in the intervention arms valued the weekly weigh-in at the commercial programme meetings and reported that this influenced their daily food choices, the measurement visits at the research centre provided further motivation. Participants described this as a ‘*compound effect*’ :

‘ [The measurement visit] *doesn’t contribute towards the weight loss but it did contribute towards attending Weight Watchers. So if you think about it, you’ve got the day-to-day stuff where “Do I, am I going to eat a cupcake?” “No, because I’ve got Weight Watchers coming up”. There’s that motivation but there’s also “Should I actually bother going to Weight Watchers? Well it takes about half an hour or more out of my time … but I’ve got the study coming up so I really should turn up. So you’ve got the two different motivators … So I think there is a compound effect there.*’

(Male, 38)

Participants also thought the 12- and 24-month measurement appointments would keep them on track once their 12/52-week programme had finished. In this sense, the follow-up measurements maintained a sense of commitment to weight loss and so, from their perspective, constituted part of the intervention.

Overall, features of trial participation meant that participants reported that attending the commercial programme during the trial was different from if they had been more usual attendees. Although this might only be conjecture on their part, it seems clear that apparently inconsequential features of the study, introduced to enable the trial to take place, nevertheless played an important symbolic role. In particular, the letter and vouchers served to authenticate the status of the participant as an NHS ‘patient’ and, where relevant, shifted their views of weight to being primarily a health issue.

## DISCUSSION

### Summary

Primary care referral to a commercial programme aligns with patients’ understanding of being overweight or obese as states that may have negative health consequences, but are ostensibly lifestyle issues requiring a non-medical solution. Despite this, participants in this trial appeared to carry their interactions with their GP and the NHS (and research staff viewed as conduits of the former) through to their experiences of the commercial programme via such symbolic elements as the GP letter and vouchers, shaping their experiences outside the NHS environment. The letter from the GP endorsed participation in the commercial programme and engendered a sense of obligation, providing motivation to attend. The weekly vouchers were experienced as a symbolic resource that maintained a link with the GP, legitimised meeting attendance as a ‘medical’ necessity rather than a luxury, and associated the programme with public funding and a sense of responsibility. Finally, the range of clinical measurements, in combination with a discussion about disease risk, provided participants with a new language through which they could acknowledge the seriousness of being overweight or obese and recognise the ‘tangible’ health benefits of weight loss. While participants acknowledged the impact that body weight had on their health, they did not view the NHS as the most appropriate space to address their weight. The clinical framing nevertheless provided a validating impetus for participants to seek support in an overtly non-clinical domain that aligned with their perception of weight loss as a non-medical ‘lifestyle’ issue.

### Strengths and limitations

A qualitative design with open-ended questions allowed participants to focus on aspects of their experience that were most important to them. Home interviews with a researcher not directly involved in the trial may have facilitated free expression. For pragmatic reasons, the current study only included participants from the Cambridge centre of the trial. However, within this centre, participants were purposely selected to maximise demographic diversity.

Although participants were informed that the trial and assessments were for research purposes and separate from both their GP and the commercial intervention, they did not always perceive these distinctions. This can be considered a limitation in the attempt to isolate the impact of the intervention. However, this ‘limitation’ is likely present but not acknowledged in most similar studies, and it is important to identify influential contextual factors outside the intervention itself. For this reason, the current study evaluated the ways in which the intervention and the trial procedures impacted patient experience in order to suggest how both aspects can be leveraged by GPs in practice.

### Comparison with existing literature

Previous studies have shown that people often distance themselves from the social stereotype of ‘obesity’ and therefore do not acknowledge the degree to which they are overweight or the health risks associated with this.^14^,^15^ This study supports previous findings that adults who are overweight or obese feel ambivalent about approaching GPs for help with weight loss and see community-based support groups as a more appropriate intervention.^3^,^5^,^6^

However, in contrast to comparative studies that reinforce the distinction between commercial and health service sectors,^5^,^16^ this study provides a more nuanced interpretation of primary care referral to commercial programmes, and shows how patients experience the partnership between the two sectors. The findings that the GP introduction by referral letter and the taking of clinical measurements at follow-up appointments may enhance the intervention experience echoes previous research that suggests ‘free’ GP referral for services which normally cost people money often encourage participation.^5^,^17^

The current study also suggests that by being able to visualise their charted measurements with regard to disease risk helps people acknowledge their current weight status and the associated health consequences, providing a new language with which they can experience a sense of progress that verbal expressions of weight alone cannot provide.

### Implications for research and practice

This study found that the letter of invitation, sent to all eligible participants in the practice, but individually addressed and signed by their GP, was interpreted as a personalised medical recommendation. The use of trial support staff to take a small number of clinical measurements and give easy-to-understand visual feedback framed the intervention around clear health goals. Patients perceived these measurements as medical monitoring because they were copied to their GP. If the main WRAP trial confirms previous findings that commercial programmes are more effective than usual primary care services,^8^,^10^ then it may be valuable to consider to what extent explicit GP and NHS linkage to commercial programmes may provide an additional ‘active ingredient’ in treating obesity. Short follow-up appointments (similar to those provided by the trial), with clinical measurements fed back to the GP, could be implemented to potentially enhance attendance, adherence, and sustain intervention effects. Further research should explore the most effective way for practitioners to conduct referrals and monitor progress in order to maximise the benefits for patients participating in commercial programmes.
